# Vertical distribution of polycyclic aromatic hydrocarbons in the brackish sea water column: ex situ experiment

**DOI:** 10.7717/peerj.10087

**Published:** 2020-10-27

**Authors:** Zilvinas Kryzevicius, Kristina Mickuviene, Martynas Bucas, Monika Vilkiene, Audrone Zukauskaite

**Affiliations:** 1 Faculty of Marine Technologies and Natural Science, Klaipeda University, Klaipėda, Lithuania; 2 Vėžaičiai Branch, Lithuanian Research Centre for Agriculture and Forestry, Vėžaičiai, Klaipėda Dist., Lithuania

**Keywords:** Oil spill, Polycyclic aromatic hydrocarbons, Vertical distribution, Suspended and dissolved phases, The concentration ratio

## Abstract

**Background:**

Oil spills can cause severe damage within a marine ecosystem. Following a spill, the soluble fraction of polycyclic aromatic hydrocarbons is rapidly released into the water column. These remain dissolved in seawater over an extended period of time, even should the insoluble fraction be removed. The vertical distribution of the aromatic hydrocarbon component and how these become transferred is poorly understood in brackish waters. This study examines the vertical distribution of polycyclic aromatic hydrocarbons having been released from a controlled film of spilled oil onto the surface of brackish water.

**Methods:**

The study was undertaken under controlled conditions so as to minimize the variability of environmental factors such as temperature and hydrodynamics. The distribution of polycyclic aromatic hydrocarbons was measured in the dissolved and suspended phases throughout the 1 m water column with different intensity of water sampling: 1, 2, 4, 7, 72, 120, 336, 504 and 984 h.

**Results:**

The total concentration of polycyclic aromatic hydrocarbons ranged from 19.01 to 214.85 ng L^–1^ in the dissolved phase and from 5.14 to 63.92 ng L^–1^ in the suspended phase. These hydrocarbons were released immediately following a controlled spill attaining 214.9 ng L^–1^ in the dissolved phase and 54.4 ng L^–1^ in the suspended phase near the cylinder bottom after 1–2 h. The 2–3 ring polycyclic aromatic hydrocarbons dominated in the dissolved phase (60–80%), whereas the greater amount of 4–6 ring polycyclic aromatic hydrocarbons (55–90%) occurred in the suspended phase. A relatively low negative correlation (r_S_ = –0.41) was determined between the concentration of phenanthrene and suspended matter, whereas a high negative correlation (*r* =  − 0.79) was found between the concentration of pyrene and suspended matter. Despite the differences in the relationships between the concentration ratio and amount of suspended matter the obtained regressions allow roughly to predict the concentration of polycyclic aromatic hydrocarbons.

## Introduction

On account of the low exchange rate of water with the North Sea, with the high residence time of approximately twenty years, makes the Baltic Sea sensitive to pollutants (*Zettler et al., 2013*). This sea has one of the greatest volumes of shipping (*Ruczyńska, Szlinder-Richert & Malesa-Ciećwierz, 2011*), where about 90% of oil and its products are being transported in Europe (HELCOM, 2018). There has been a decline of large oil spills by shipping since 2000 due to improvements in ship safety and management. During 2017 there were two spills of >700 Mt and four in the range of 7–700 Mt. Although the actual number of small spills is not fully known (ITOPF, 2018). Such spills release a significant amount of hydrocarbons and their derivatives. However, there are also other sources from incomplete combustion of fossil organic matter and fossil fuel (*Li & Chen, 2003; Saha et al., 2009; Eide et al., 2011; Kuo et al., 2011*). Oil spills cause damage within the marine ecosystems by impacting the populations of sensitive species, reducing sediment quality and causing impacts to aquaculture and fisheries (*Baksh et al., 2018; Khan et al., 2018*).

Polycyclic aromatic hydrocarbons (PAHs) are toxic and carcinogenic compounds which occur in crude oil (*Page et al., 2002; Kottuparambil & Agusti, 2018*). The soluble fraction of PAHs is rapidly released following a spill and remain dissolved in seawater for an extended period of time, even should the insoluble fraction be removed (*Gonzalez et al., 2009; Eide et al., 2011*). Their subsequent breakdown can lead to the formation of even more toxic compounds such as nitrated PAHs some of which are considered to have a higher mutagenicity (*Topinka et al., 1998*). PAHs can affect marine organisms. This depends on their concentration in the dissolved phase because dissolved organic matter is more readily available to biota (*Mott, 2002*).

Since PAHs have a relatively low solubility in water they are preferentially associated with particles in the coastal marine waters, which settle-out as sediments (*Hatzianestis & Sklivagou, 2002; Karlsson & Viklander, 2008; Guigue et al., 2014*). However, separate studies Bouloubassi & Saliot (1991) and Tedetti et al. (2011) found concentrations of PAHs in the dissolved phase were similar or even greater than those recorded in the particulate phase. This means there is a lack of systematic assessment of PAHs between the dissolved and suspended phases on account of the many transformation processes during their occurrence in the water column of such oil products.

Most studies (*Wang, Shen & Zheng, 2005; Wang et al., 2008; Zadeh & Hejazi, 2012*) have focused on the horizontal modelling of oil spills and the transfer processes that take place on the water surface (*Wang & Zhang, 2012; Pilzis, Vaisis & Romagnoli, 2017; Long et al., 2018*). The vertical distribution of hydrocarbons and processes of oil spill transfer is less well known.

This study evaluated the vertical distribution of PAHs in brackish water under experimental conditions. We targeted individual PAHs released from the surface oil and the spatial differences of PAHs in the dissolved and suspended phases. For prediction of distribution of PAHs in different phases, the relationships between the concentration ratio and the amount of suspended matter were tested.

## Materials & Methods

### Experimental design

The research was carried out under laboratory conditions (Klaipeda University) during 2017–2018. An oil spill was imitated in the polyvinylchloride cylinders (height—1.1 m, diameter—0.2 m) with infusive systems for vertical water sampling. Three cylinders were filled up with 34 L of brackish water of the Baltic Sea (salinity –6.2, temperature 14 °C) and with 50 g of crude oil (its density at 20 °C—0.8665 kg dm^−^^3^). For the control, three other cylinders were filled up with natural brackish water. The cylinders were sealed with a transparent methacrylate plate. Water chemical composition in the cylinders was monitored over a period of 40 days. The samples of 50 mL of water were taken from 2 layers of water-column in the cylinder: (1) surface (∼5 cm under the film of crude oil) and (2) near-bottom (∼5 cm above the bottom). Different intensity of water sampling was chosen to find short-term patterns (ranging from hours immediately after the spill to days and weeks) in a release of petroleum hydrocarbons from an oil film: 1 h, 2 h, 4 h, 7 h, 72 h (3 days), 120 h (5 days), 336 h (14 days), 504 h (21 days) and 984 h (41 days).

### Chemical analysis

The water samples were filtrated through glass fibre filters (Whatman GF/F with 0.7 µm effective pore size; precombusted at 450 °C for 5 h). The GF/F filters were placed in pre-cleaned glass dishes, wrapped with aluminium foil and stored at − 18 °C until extraction. Then, GF/F filters were dried and weighed to calculate a content of suspended matter. The GF/F filters with trapped particulate matter were then extracted by hexane/dichloromethane (Sigma Aldrich, CHROMASOLV^®^, ≥95%) (1:1, v/v) in an ultrasonic bath (Ultrasonic baths Sonorex Digitec, Type DT 100) for three times (each time for 30 min). The three extracts were all combined and transferred to a flask. The extract was concentrated to nearly dry by rotary evaporation (IKA RV–10 Digital), then solvent exchanged into hexane around one mL and was analyzed afterwards.

The filtrated water samples (dissolved organic matter) were extracted 3 times using the mixture of 20 mL of hexane and dichloromethane (1:2). Collected extracts were transferred through analytically pure anhydrous sodium sulphate (Sigma Aldrich, anhydrous, Redi-Dri^®^, ACS reagent, ≥ 99%). The extract was concentrated to nearly dry by rotary evaporation, then solvent exchanged into hexane around one mL. The extracts were cleaned up using a five mL 2:3 (v/v) alumina:silica gel chromatography column. PAHs were eluted with 10 mL of n-hexane/dichloromethane (1:1 v/v). The fractions were concentrated to one mL under a stream of pure nitrogen and stored at 4 °C prior to instrumental analysis.

A concentration of PAHs was determined using the gas chromatograph Shimadzu GC-2010 Plus with the Flame-Ionization Detector (GC-FID) and the Shimadzu 7683 Auto-sampler. The chromatograph was calibrated with Polynuclear Aromatic Hydrocarbons Mix Analytical Standard (Supelco, 48905-U, 16 compounds), 2000 µg mL^−1^ each component in methylene chloride: benzene (1:1). The PAHs with 2–6 aromatic rings were detected: Naphthalene (Naph), Phenanthrene (Phe), Anthracene (Ant), Fluoranthene (Flt), Pyrene (Pyr), Chrysene (Chr), Benzo[k]fluoranthene (BkF), Benzo[a]anthracene (BaA), Benzo[a]pyrene (BaP), Indeno[1,2,3-c,d]pyrene (IndP) and Benzo[ghi]perylene (BP), Acenaphthene, Acenaphthylene, Benzo[*b* ]fluoranthenem, Dibenz[*a*,*h*]anthracene, Fluorene. The chromatography column: Rxi^®^ –1ms, Crossbond^®^  100% dimethylpolysiloxane, length – 20 m, diameter – 0.18 mm, 0.18 um df. Temperature was set up from 55 to 300 °C at a rate of 10 °C per minute and was maintained at 300 °C for 15 min. Carrier gases was helium (0.99 mL min^−1^). The detector temperature was 320 °C. Each concentration of PAHs was measured 3 times. A total concentration of PAHs was obtained by sum of individual concentrations of PAHs.

A concentration ratio was determined as proportion of compounds associated with suspended particles phase (Cs) and compounds in a dissolved phase (Cw): concentration ratio = Cs/Cw.

### Statistical analysis

Generalized additive models were used to estimate temporal trends in the total concentrations of PAHs. Generalized additive models are able to model non-linear relationships between time and a response variable and can handle the irregular spacing in time series (*Simpson, 2018*). Thin-plate regression splines with at least 3 degrees of freedom and the gamma value of 1.4 were used to parametrise the smooth functions of time (Wood & Augustin, 2002). The estimated degree of freedom (edf), F and *P* values were provided only for significant temporal trends. Values of edf ≤ 1 indicate linear trend, whereas values of edf > 1indicate nonlinear trends. Generalized additive models were performed using the mgcv package (Wood, Pya & Säfken, 2016) in R 3.4.4 (R Core Team, 2020; RStudio Team, 2020).

The effects of layers (near-bottom and surface), phases (dissolved and suspended) and time of sampling on concentrations of PAHs were tested with generalized least squared models because of violation of the homogeneity of variance assumption. The full model with all factors (predictors) was compared with models allowing different variance structures according to the type of predictors using the Akaike’s information criteria (*Zuur et al., 2009*). The backward selection of significant predictors was performed comparing the full model with reduced model. The generalized least squared models were implemented with nlme package (*Pinheiro et al., 2020*) in R.

The multiple, all pairwise comparisons of means between the treatments (layers, phases and time) were tested by the Games-Howell post hoc test due to violation of the homogeneity of variance assumption. The test was performed with the PMCMRplus package (*Pohlert, 2019*) in R.

Depending on how data fit a normal distribution, the Pearson product-moment correlation coefficient (r) or the Spearman’s rank correlation coefficient (r_*S*_) was determined between the concentration ratio for Phenanthrene and Pyrene and the amount of suspended matter. Simple linear regression models were fitted for both relationships using the lm function in R and coefficients of determination (r^2^) were determined. The results of all statistical tests were regarded significant with *P* < 0.05.

## Results

The PAHs with 2–6 aromatic rings were determined: Naphthalene (Naph), Phenanthrene (Phe), Anthracene (Ant), Fluoranthene (Flt), Pyrene (Pyr), Chrysene (Chr), Benzo[k]fluoranthene (BkF), Benzo[a]anthracene (BaA), Benzo[a]pyrene (BaP), Indeno[1,2,3-c,d]pyrene (IndP) and Benzo[ghi]perylene (BP).

**Figure 1:**
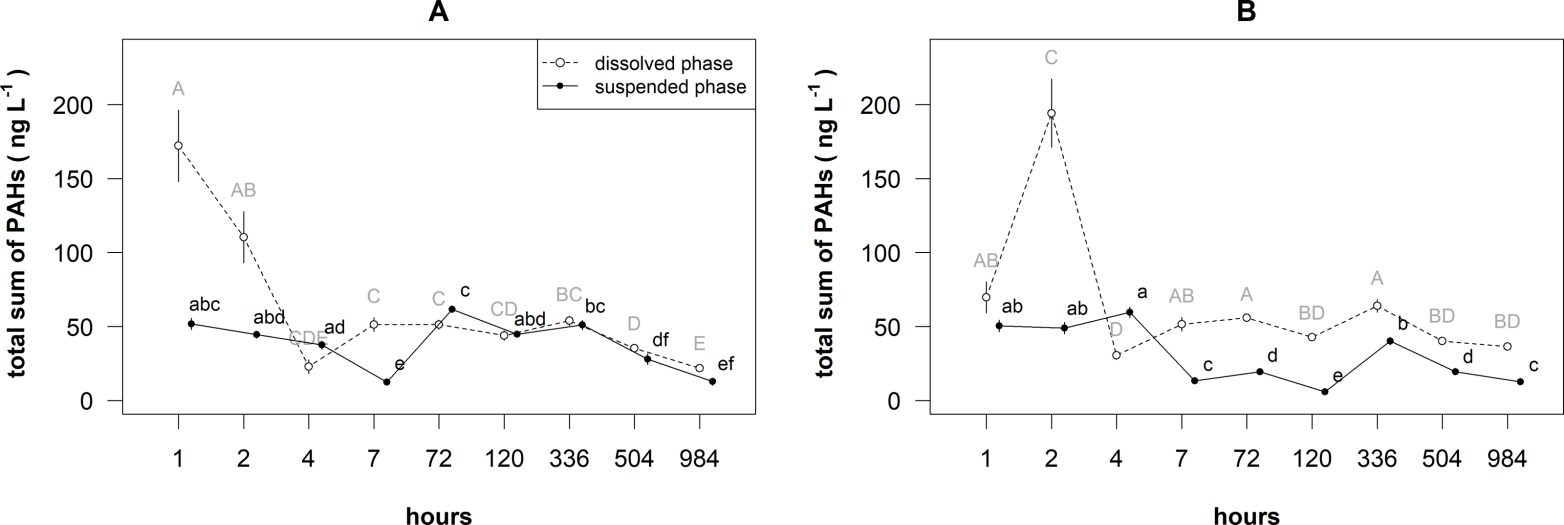
Temporal patterns of the mean (±standard deviation) concentration of total PAHs in the dissolved and suspended phases near the surface (A) and bottom (B) layers of the cylinders. Different letters indicate significant differences among the means (*P* < 0.05, Games-Howell post-hoc test), where capital letters stand for dissolved phase, and small letters stand for suspended phase.

Linearly decreasing trends of the total concentrations of PAHs (Fig. 1 and Table 1) were recorded in the dissolved phase of surface layer and both phases of bottom layers, while a nonlinear (i.e., bell-shaped) decreasing trend was found in the suspended phase of surface layer.

**Table 1:** Statistical significance of trends in the total concentrations of PAHs in different layers and phases.

**Layer**	**Phase**	**Estimated degree of freedom**	*F* value	*P* value
Surface	Dissolved	1.015	6.112	<0.001
Surface	Suspended	3.584	21.750	<0.001
Bottom	Dissolved	0.87	1.86	<0.01
Bottom	Suspended	0.848	2.373	<0.005

The mean of total sum of PAHs concentrations significantly differed between the layers, phases and time (i.e., statistically significant their interaction: *F* = 43.694, *df* = 1, *P* < 0.001). In the surface layer, the total concentration of PAHs ranged from 19.01 to 198.19 ng L^−1^ in the dissolved phase (Fig. 1), whereas in the suspended phase it was from 10.05 to 63.92 ng L^−1^. The concentration of PAHs in this phase ranged 37–68% from the total amount of PAHs. In the bottom layer, the total concentration of PAHs ranged from 28.29 to 214.85 ng L^−1^ in the dissolved phase, whereas in the suspended phase it was from 5.14 to 63.24 ng L^−1^.

In the surface layer, the means of total sum of PAHs concentrations in the dissolved phase after 1 and 2 h were significantly (*P* < 0.05) higher than later mean and the means in the suspended phase (Fig. 1A). In the dissolved phase, the means of total sum of PAHs concentrations after 984 h was significantly lower than the means after 4–504 h. In the suspended phase, the means of total sum of PAHs concentrations after 7,504 and 984 h were significantly lower than the other means.

In the bottom layer, the mean of total sum of PAHs concentration in the dissolved phase after 2 h was significantly (*P* < 0.05) higher than after other hours and the means in the suspended phase (Fig. 1B). In the dissolved phase, the mean of total sum of PAHs concentration after 4 h was significantly lower than the means after 1–2, 7–72 and 366 h. In the suspended phase, the means of concentrations after 1–4 h were significantly higher than the means after other hours.

**Figure 2:**
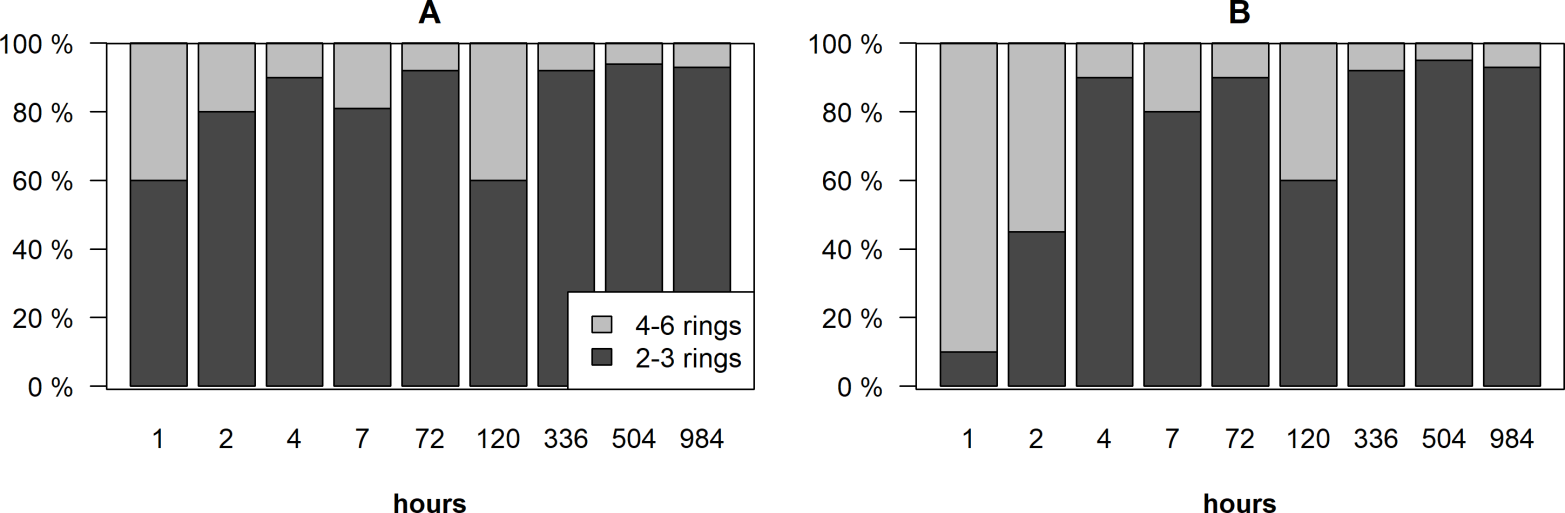
Distribution of 2–3 rings and 4–6 rings PAHs in the dissolved phase (A) and in the suspended phase (B) in the surface and bottom layers of the cylinders.

The biggest difference in the distribution of PAHs was found during the first two hours after the spill, especially in the suspended phase (Fig. 2). In the dissolved phase, 2–3 rings PAHs dominated (60–80%), while the highest amount of 4–6 rings PAHs (55–90%) were recorded in the suspended phase. Two hours after the spill, the amount of 2–3 rings PAHs increased more than 80% in the dissolved and the suspended phases and dominated in both phases.

**Figure 3:**
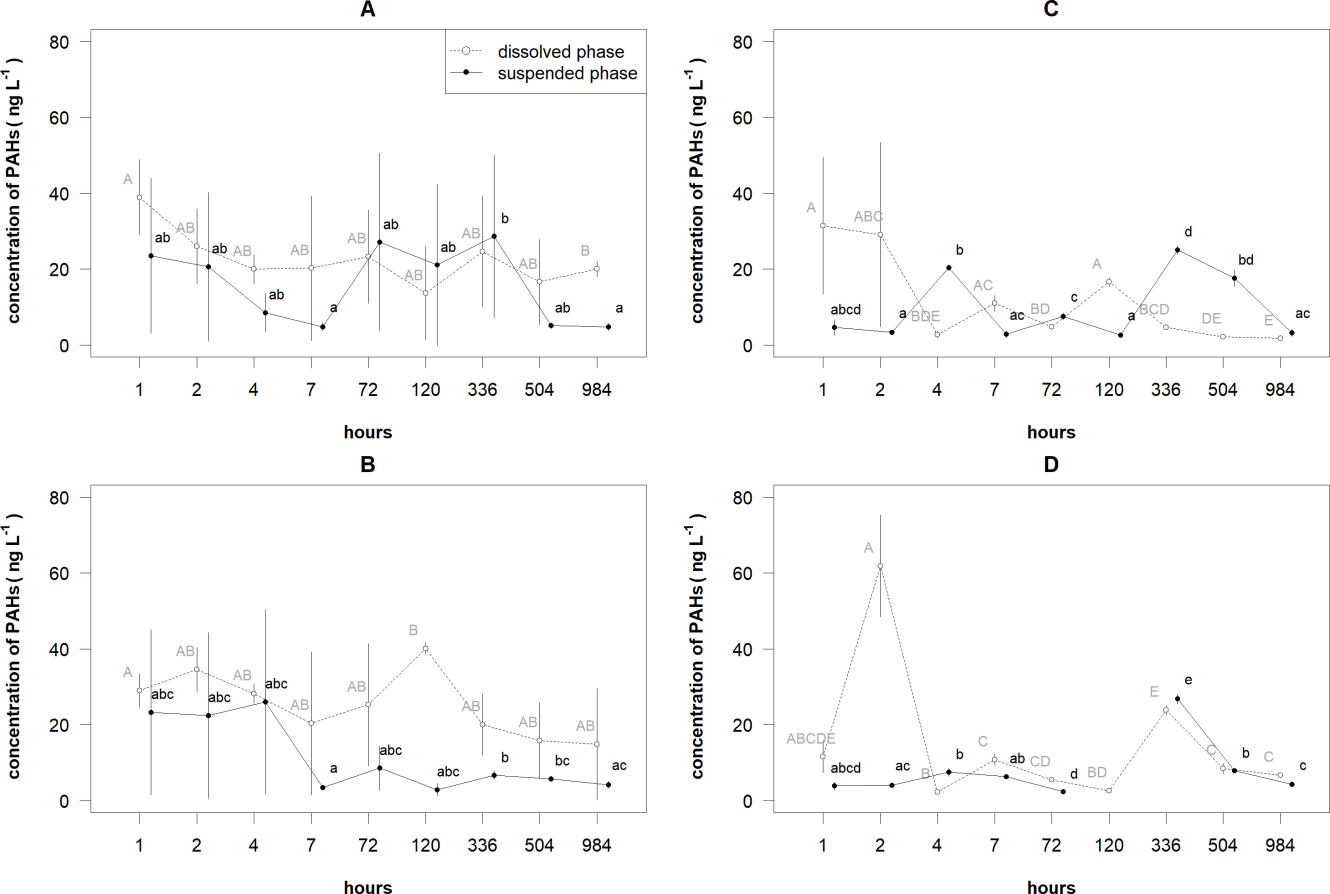
The mean (±standard deviation) concentrations of 2–3 rings and 4–6 rings PAHs in the dissolved and suspended phases near the surface and bottom layers of the cylinders. The 2–3 rings PAHs near the surface (A) and bottom (B) layers of the cylinders and 4–6 rings PAHs phases near the surface (C) and bottom (D) layers of the cylinders.

The similar patterns were observed in the changes of the mean concentrations of 2–3 and 4–6 rings PAHs in different layers and phases (Fig. 3). For 2–3 rings PAHs, decreasing trends of the total concentrations of PAHs were determined in the surface layer for suspended phase and in the bottom layer for both phases (Table 2). For 4–6 rings PAHs, decreasing trends of the total concentrations of PAHs were determined in the surface layer for dissolved phase and in the bottom layer for both phases. A bell-shaped trend was estimated in the surface layer for suspended phase.

**Table 2:** Statistical significance of trends in the total concentrations of 2–3 and 4–6 rings PAHs in different layers and phases.

**Rings**	**Layer**	**Phase**	**Estimated degree of freedom**	*F* value	*P* value
2–3	Surface	Dissolved	<0.001	0.000	0.226
2–3	Surface	Suspended	0.912	3.272	<0.001
2–3	Bottom	Dissolved	0.86	1.94	<0.01
2–3	Bottom	Suspended	1.617	4.410	<0.005
4–6	Surface	Dissolved	3.01	2.83	<0.05
4–6	Surface	Suspended	1.009	10.490	<0.001
4–6	Bottom	Dissolved	0.78	1.54	<0.01
4–6	Bottom	Suspended	3.987	147.300	<0.001

The mean concentrations of 2–3 and 4–6 rings PAHs significantly differed between the layers, phases and time (i.e., their statistical interactions respectively for 2–3 and 4–6 rings PAHs: *F* = 9.067, *df* = 1, *P* < 0.005 and *F* = 26.834, *df* = 1, *P* < 0.001). For 2–3 rings PAHs in the surface layer, only the mean concentration of PAHs in the dissolved phase after 1 h was significantly (*P* < 0.05) higher than the one after 984 h (Fig. 3A). In the suspended phase, only the mean concentration of PAHs after 336 h was significantly higher than the means after 7 and 984 h. In the bottom layer, the mean concentration of PAHs in the dissolved phase after 120 h was significantly higher than the one after 1 h (Fig. 3B. The mean concentration of PAHs in the suspended phase after 7 h was significantly lower than the means after 336 and 504 h; the mean after 336 h was significantly (*P* < 0.05) higher than the one after 984 h.

For 4–6 rings PAHs in the surface layer, the mean concentration of PAHs in the dissolved phase after 1 h was significantly (*P* < 0.005) higher than the means after all hours, except after 2, 7 and 120 h (Fig. 3C). In the same layer, the mean concentrations of PAHs in the suspended phase after 4, 336 and 504 h were significantly (*P* < 0.05) higher than the other means . In the bottom layer, the mean concentration of PAHs in the dissolved phase after 2 h was significantly (*P* < 0.001) higher than the other means, except after 1 h, which did not significantly differ from the rest means. In the same layer, the mean concentration of PAHs in the suspended phase after 336 h was significantly (*P* < 0.05) higher than the other means, except the one after 504 h.

**Figure 4:**
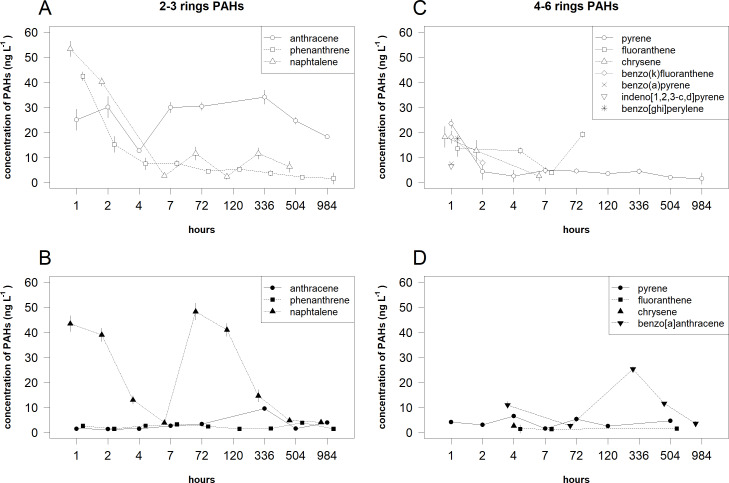
Temporal patterns of the mean (±standard deviation) concentrations of 2–3 rings and 4–6 rings PAHs compounds in the dissolved phase and suspended phase. The 2–3 rings PAHs in the dissolved (A) and suspended (B) phases and 4–6 rings PAHs in the dissolved (C) and suspended (D) phases.

For 2–3 rings PAHs in the dissolved phase, the decreasing trends of Phe and Naph concentrations were determined (Fig. 4 and Table 3). In the same phase, a bell-shaped trend was recorded for Ant concentrations. In the suspended phase, nonlinear trends were determined for different PAHs compounds: a sharp decrease up to 7 h and a bell-shaped trend later for Naph, a bell-shaped trend for Phe and Ant concentrations.

**Table 3:** Statistical significance of trends in the total concentrations of 2–3 and 4–6 rings PAHs compounds in different phases.

**Rings**	**Compound**	**Phase**	**Estimated degree of freedom**	*F* value	*P* value
2–3	Ant	Dissolved	2.58	2.43	<0.05
2–3	Ant	Suspended	3.957	127.2	<0.001
2–3	Naph	Dissolved	3.763	15.52	<0.001
2–3	Naph	Suspended	26.022	1.664	<0.001
2–3	Phe	Dissolved	0.834	2.918	<0.01
2–3	Phe	Suspended	3.514	6.513	<0.001
4–6	BkF	Dissolved	1.00	41.17	<0.01
4–6	Chr	Dissolved	0.970	12.530	<0.001
4–6	Flt	Dissolved	2.818	34.24	<0.001
4–6	Flt	Suspended	<0.001	0.000	0.423
4–6	Pyr	Dissolved	5.205	0.753	<0.001
4–6	Pyr	Suspended	0.61	0.56	0.06
4–6	BaA	Suspended	3.994	250.500	<0.001

For 4–6 rings PAHs in the dissolved phase, the decreasing trends of Pyr, Chr and BkF concentrations were determined (Fig. 4 and Table 3). BaP, IndP and BP were detected only after 1 h. In the same phase, a U-shaped trend was recorded for Flt concentrations. In the suspended phase, only a bell-shaped trend was determined for BaA concentrations. Chr was detected only after 4 h.

For 2–3 rings PAHs, the mean concentrations of toxic compounds (Ant, Phe and Naph) significantly differed between the phases and time (i.e., their statistical interactions respectively: *F* = 472.486, *df* = 1, *P* < 0.001; *F* = 47.712, *df* = 1, *P* < 0.001 and *F* = 43.919, *df* = 1, *P* < 0.001). The mean concentrations of Ant in the dissolved phase were significantly higher (*P* < 0.001) than the one in the suspended phase (Fig. 4). In the dissolved phase, the mean concentration of Ant after 4 h was significantly (*P* < 0.05) lower than the means after 2 and 7-984 h. In the suspended phase, the mean concentrations of Ant after 336 h were significantly (*P* < 0.05) higher than the means 1–72 h. The mean concentrations of Phe in the dissolved phase were significantly higher (*P* < 0.05) than in the suspended phase only after 1 and 2 h. In the dissolved phase, the mean concentrations of Phe after 1 and 2 h were significantly (*P* < 0.05) higher than the means later. The mean concentrations of Naph in the dissolved phase were significantly higher (*P* < 0.05) than the one in the suspended phase only after 72 h. In the dissolved phase, the mean concentration of Naph after 1 h was significantly (*P* < 0.05) higher than the means after 2, 7 and 336 h. In the suspended phase, the means concentration of Naph after 1 and 2 h were significantly (*P* < 0.05) higher than the means after 4, 7, 336 and 984 h.

For 4–6 rings PAHs, the mean concentrations of toxic compounds (Pyr and Flt) significantly differed between the phases and time (i.e., their statistical interactions respectively: *F* = 28.620, *df* = 1, *P* < 0.001 and *F* = 46.488, *df* = 1, *P* < 0.001). The mean concentration of Pyr in the dissolved phase was significantly higher (*P* < 0.001) than the one in the suspended phase only after 1 h (Fig. 4). In the dissolved phase, the mean concentration of Pyr after 1 h was significantly (*P* < 0.05) higher than the means after 7-504 h. In the same phase, the mean concentration of Flt after 7 h was significantly (*P* < 0.05) lower than the means after 1, 4 and 72 h. In the suspended phase, the mean concentration of Pyr after 7 h was significantly (*P* < 0.05) lower than the means after 1–4 and 504 h.

**Figure 5:**
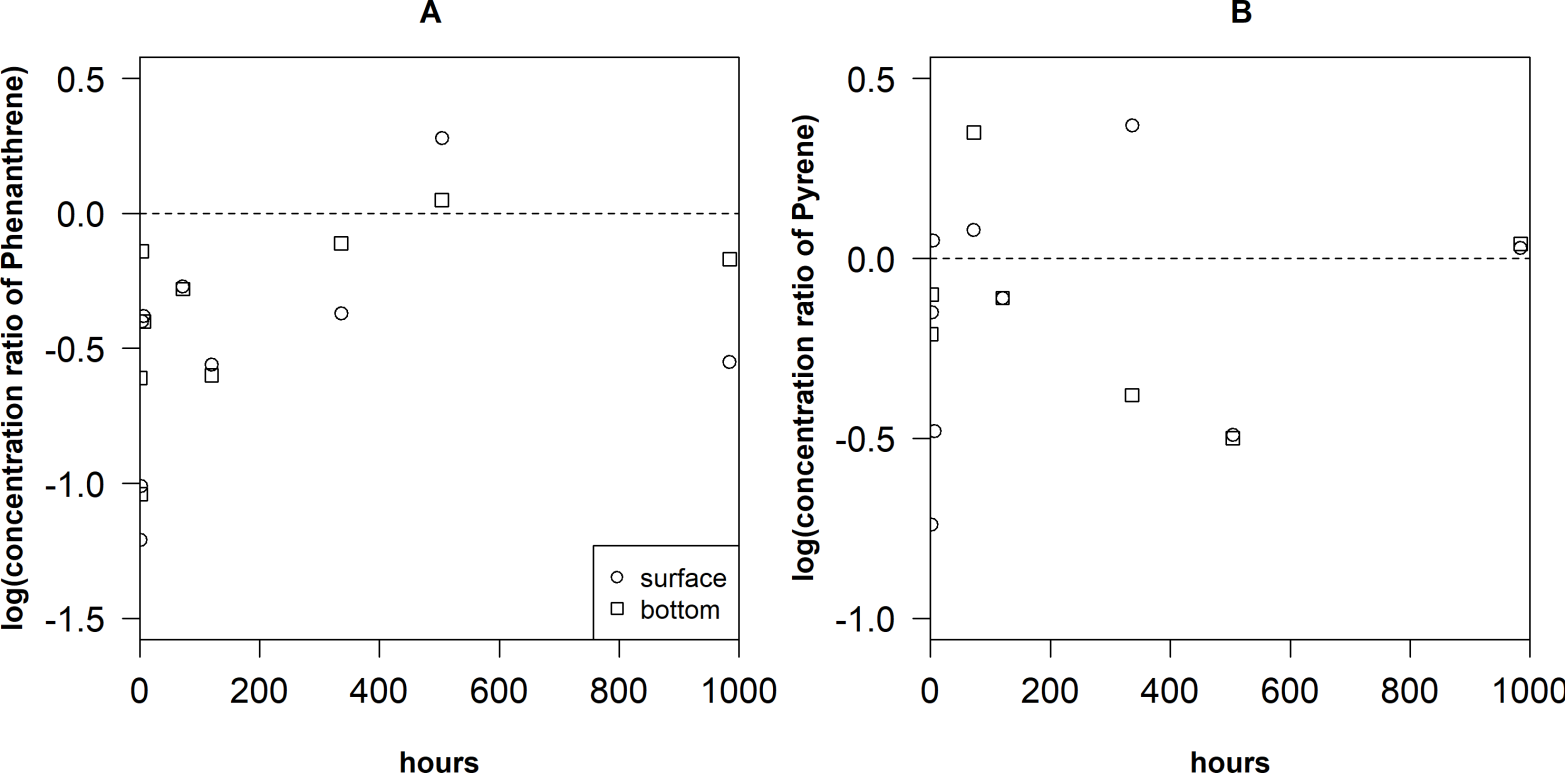
Variation of concentration ratio of Phenantrene (A) and Pyrene (B) in the surface and bottom layers of the cylinders. The dotted line represents 0 for *y* axis.

The sediment-water concentration ratio of Phe an Pyr (Fig. 5) were the lowest on the surface during the first hours after the spill (−1.21–(−1.01) and −0.61–(−0.49), respectively). After 4 h, Pyr reached the highest values of concentration ratio (0.87) near the bottom layers. Later, the concentration ratio values of Phe and Pyr decreased until they get again high in the samples taken after 504 h in the surface layer (0.28 and 0.37, respectively).

**Figure 6:**
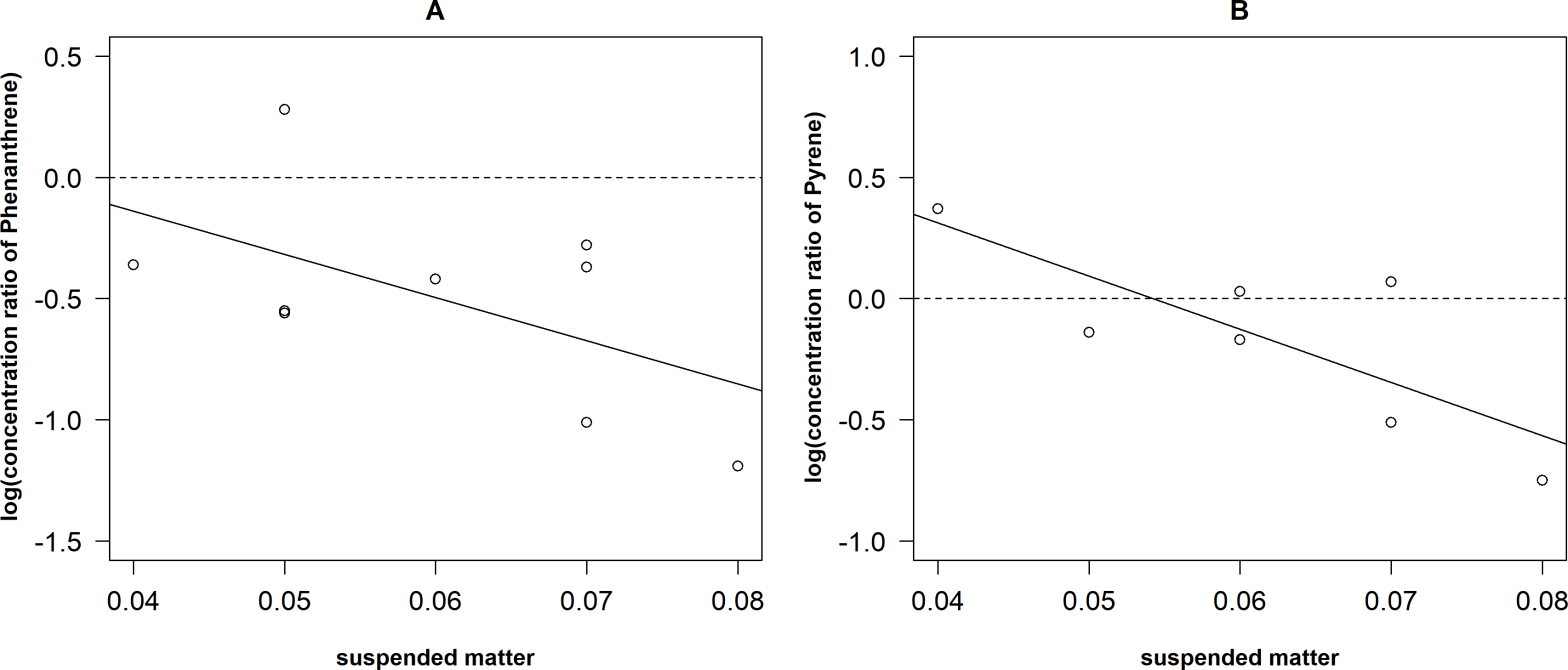
Relationships between the concentration of ratio of Phenantrene (A) and Pyrene (B) and the amount of the suspended matter. The line –fitted regression model.

There was low negative correlation between concentration ratio of Phe and the concentration of suspended matter (Fig. 6) but it was not significant (r_*S*_ = −0.41, *P* = 0.27, *N* = 9). However, concentration ratio of Pyr significantly negatively correlated with the concentration of suspended matter (*r* =  − 0.79, *P* < 0.05, *N* = 7). The linear regression models were fitted for both relationships: log(y_*Pyr*_) = 1.19–21.95*suspended matter (r^2^ = 0.62) and log(y _*Phe*_) = 0.57–17.79*suspended matter (r^2^ = 0.21).

## Discussion

In this study over the forty-one days we found a significant variation in the dissolved and suspended phases of PAHs throughout the water column. Since PAHs tend to be hydrophobic most of these were found in the suspended phase (Tedetti et al., 2011; Chiffre et al., 2015). However, in our study, there was the significantly lower mean concentration of PAHs in the suspended phase when compared with the dissolved phase. This could be explained should the colloidal phase, including small particles, was not trapped within the filters (Brown & Peake, 2003). In this case, the concentration of PAHs in the dissolved phase was higher than what has been recorded in the literature (*Dachs et al., 2002; Huang et al., 2017*). This could be due to the analyzed distribution of PAHs in the natural environment, where water is mixed both vertically and horizontally (by wind and tidal vectors).

In our study, the release of PAHs from the surface film followed immediately after the spill (Fig. 1). This is in agreement with results from other studies (*Gonzalez et al., 2009; Eide et al., 2011*), where PAHs soluble fraction is rapidly released into the water column and the PAHs remain dissolved in seawater over an extended period of time, even should the insoluble fraction have been removed. The mean concentrations of PAHs in our study did not significantly change throughout the water column after 1–2 h. It was some time later (after 4–7 h) before the release of PAHs from the film slowed. This could have been due to absorbed PAH component having either precipitated onto the bottom layer (Payne, Clayton & Kirstein, 2003) or attached to particles (Karlsson & Viklander, 2008).

With respect to the temporal changes of the mean concentrations of PAHs, there were recorded several exceptional concentrations (including relatively low standard deviations) compared to the nearest estimates, especially near the bottom layer, e.g., for 2–3 rings PAHs, after 120 h in the dissolved phase; for 4–6 rings PAHs, after 336 h in both phases (Fig. 3). Most probably, the overestimations of the mean concentrations resulted from the sampling error affected by bottom of cylinder.

In general, the mean concentrations of PAHs near the bottom layer were significantly higher in the dissolved phase than in the suspended one for 2–3 and 4–6 rings PAHs. These differences can be explained by the fact that a large part of PAHs in the dissolved phase is in the colloidal form or in very small particles, which could not be trapped by the filter as suspended particles (*Brown & Peake, 2003*). If the balance between the dissolved and suspended phases is disturbed, PAHs can desorb from the suspended phase into the dissolved one (*Witt, 2002*). This can explain why the concentrations of 2–3 rings PAHs in the suspended phase increased in the samples taken after 72–336 h (Fig. 3). However, *Fernandes et al. (1997)* showed that PAHs associated with the suspended phase do not release into the dissolved phase but can be found as occluded or strongly bound with fined-grained particles, which cannot be trapped by filters.

The PAH compounds are very toxic and their presence in the environment can be considered as an ecological indicator. For instance, a concentration of Naph is particularly important for toxicity, and together with other low-molecular-weight PAHs are monitored for ecosystem recovery after oil spills (*Page et al., 2002; Boehm & Page, 2007*). In this study, the temporal distribution patterns of 2–3 rings PAHs, Naph, Phe and Ant made 61% of all PAHs found in the dissolved phase during the 1st hour. Similar results were also recorded during other studies (Olivella, 2006; Deyme et al., 2011). In the dissolved phase, the concentrations of these compounds were significantly higher compared to the suspended phase (Fig. 4). It was found that the concentration of Naph in the dissolved phase was relatively high during the first two hours after the spill. This is in agreement with the results from another experiment, where Naph dominated in the water column and was the most detectable in the dissolved phase (*Luo et al., 2004*). During the first hours in our study, Naph was also dominant (84%) in the suspended phase (Fig. 4). This indicates that the oil spill happened not long ago and the oil film was not affected by noticeable weathering processes (*Elordui-Zapatarietxe et al., 2010*). In the suspended phase, relatively high concentrations of this compound were detected until 336 h from the spill and only the traces of this compound were recorded later. Dominance of Naph in PAHs can also depend on the content of hydrocarbons in the crude oil (*Gonzalez et al., 2009*).

The concentrations of 4–6 rings PAHs in the dissolved phases could be reduced throughout the study period due to the low content of these compounds in the crude oil. Another reason for this could be the ongoing oxidation processes of 4–6 rings PAH compounds.

In this study considering Kow, low-molecular-weight PAHs dominated in the dissolved phase (72 ± 23%), which confirms the results obtained by *Stortini et al. (2009)*. High concentrations of high-molecular-weight PAHs in the suspended phase were found only after few hours from the spill (Fig. 4).

The changes in distribution of concentration ratio of Phe and Pyr after 504 h (Fig. 5) can be explained by the fact that balance was disturbed by newly released PAHs from the oil film. Due to it, distribution is always in the non-equilibrium phase. It can also be noticed that concentration ratio values of Pyr in the near bottom layer were higher than that of Phe. These data are in agreement with the observed trend that hydrophobic compounds tend to associate more with suspended particles (*Witt, 2002; Wu et al., 2011*).

There were differences in the relationships between concentration ratio and the amount of the suspended matter (Fig. 6). A moderate negative correlation (*r* =  − 0.79) was determined between Pyr and the concentration of suspended matter. This could be explained by the surface layer as hydropofobicity of Pyr, which is higher than that of Phe. It leads to higher potential to interact with the suspended matter and conditions to remove PAHs from the water column (*Weber et al., 2006*). In natural environment, pollutant sorption to suspended matter influences their toxicity, bioavailability and further fate, which can be identified as key mechanism of PAHs accumulation in sediments (*Qiao, Huang & Wang, 2008*). Despite the differences in the relationships between the concentration ratio and amount of suspended matter we obtained linear regressions that allow roughly to predict the concentration of PAHs from the concentration of suspended matter. The relatively low explained variance in the concentration ratio of Phe is most likely due to low number of observations (7); however, the general trends of both regressions (i.e., beta coefficients) were similar.

## Conclusions

Experimentally it was found that the release of PAHs from the film had immediately started after the spill. The highest concentrations of PAHs were detected 214.85 ng L^−1^ in the dissolved phase and 63.92 ng L^−1^ in the suspended phase after 1 h. This highlights the amount of release of PAHs before the start of liquidation works after the oil spill. The concentration of PAHs in the dissolved phase ranged from 36.8 to 68.0% of all PAHs amount, showing that it can strongly be influenced by PAHs transfer and bioavailability.

The 2–3 rings PAHs dominated (45.0–94.0%) in the dissolved phase and were released during whole period of the study. The 4–6 rings PAHs were found in lower concentrations (in some samples < 6.0%) in the dissolved phase. In the suspended phase, higher concentrations of high-molecular-weight PAHs were found only after a few hours from spill. It can also be noticed that concentrations of PAHs near the bottom layer were significantly higher in the dissolved phase than in the suspended phase for 2–3 and 4–6 rings PAHs.

The concentration ratio of Phe and Pyr varied throughout the experiment, showing that the balance between the dissolved and suspended phases had not stabilized in the water column after oil spills. Moreover, low values of concentration ratio showed that the major part of PAHs was distributed in the dissolved phase. Despite the differences in the relationships between the concentration ratio and amount of suspended matter the obtained regressions allow roughly to predict the concentration of polycyclic aromatic hydrocarbons.

##  Supplemental Information

10.7717/peerj.10087/supp-1 Supplemental Information 1 Study data

## Additional Information and Declarations

### Data Availability

The following information was supplied regarding data availability: The raw measurements are available as a Supplemental File.
